# Dog Footprint in the Heart

**Published:** 2016-10-03

**Authors:** Hassan Aghajani, Shahrooz Yazdani, Seyed Khalil Forouzan Nia, Hamed Vahidi, Faezeh Aghajani

**Affiliations:** 1 *Tehran Heart Center, Tehran University of Medical Sciences, Tehran, Iran.*; 2 *Shahid Rajaei Hospital Medical Center, Alborz University of Medical Sciences, Karaj, Iran.*; 3 *Tehran University of Medical Sciences, Tehran, Iran.*

**Keywords:** *Echinococcosis*, *Heart*, *Female*

## Abstract

Cardiac manifestations of the hydatid cyst are relatively uncommon. Cardiac involvement may lead to the compression of vital organs, pulmonary hypertension, pericardial effusion, and even anaphylaxis. A 45-year-old woman presented to the Emergency Department of Tehran Heart Center with chest pain. Cardiac examination revealed relatively muffled heart sounds. Echocardiography demonstrated a round echolucent well-defined mass (47 × 25 mm) on the base and the mid lateral wall of the left ventricle (LV) without septation. Computed tomography angiography and cardiac magnetic resonance imaging revealed a large (52 mm) exophytic mass originating from the lateral wall of the LV with upward growth between the left anterior descending artery (LAD) and the left circumflex artery with no LV cavity obliteration. Coronary angiography showed upward displacement in the LAD with significant compressive narrowing. The patient underwent mass resection and grafting of the LAD. During surgery after the incision of the pericardium, the hydatid cyst entity of the mass was revealed. Hydatid cysts covered the anterolateral surface of the LV with adhesion to the pericardium. The patient recovered from the surgery uneventfully. Pathology report and immunological assays confirmed the diagnosis. During a 6-month postoperative follow-up period, she remained asymptomatic with complete recovery and no recurrence.

## Introduction

Hydatid disease is a parasitic infestation caused by *Echinococcus granulosus*. It is endemic in southern Europe and the Middle East.^1^ The liver and lungs are the sites most commonly involved.^2^ This parasite invades the heart rarely (in about 0.5% to 2% of all cases), and isolated cardiac involvement is even more infrequent.^3^^, ^^4^ Cardiac involvement remains clinically silent for a long time before presentation, and it becomes clinically noticeable when the cysts grow and create a pressure effect or rupture. Large cysts can compress the adjacent structures such as the pulmonary and coronary arteries. According to the literature, hydatid disease in rare cases can mimic acute myocardial ischemia.^5^^-^^8^

In this article, we present a case of hydatid cysts compressing the left anterior descending artery (LAD) with secondary significant stenosis and signs of myocardial ischemia.

## Case Report

A 45-year-old woman presented to the Emergency Department of Tehran Heart Center with chest pain. She had atypical chest discomfort of 3 months’ duration before admission, with the pain having recently exacerbated. The chest pain had initially not been exertional; nevertheless, in the days leading up to her admission, she had experienced worsening of the pain with minimum physical activity in addition to vague rest pain. Except for the chest pain and mild exertional dyspnea, however, she had no further complaints. She was a nonsmoker, and her past medical history was unremarkable: She had no known cardiac risk factors, no family history of cardiac disease of note, and no chronic medical diseases. Additionally, she did not consume any regular drugs except for some infrequent over-the-counter drugs. 

**Figure 1:**
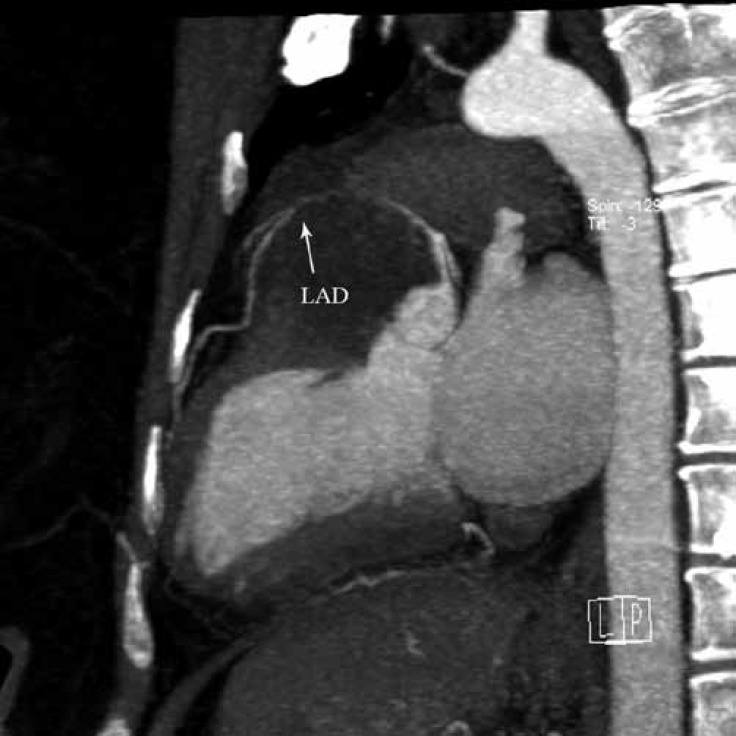
Computer tomography angiography of the patient shows that the LAD is compressed and displaced upward (arrow).

**Figure 2:**
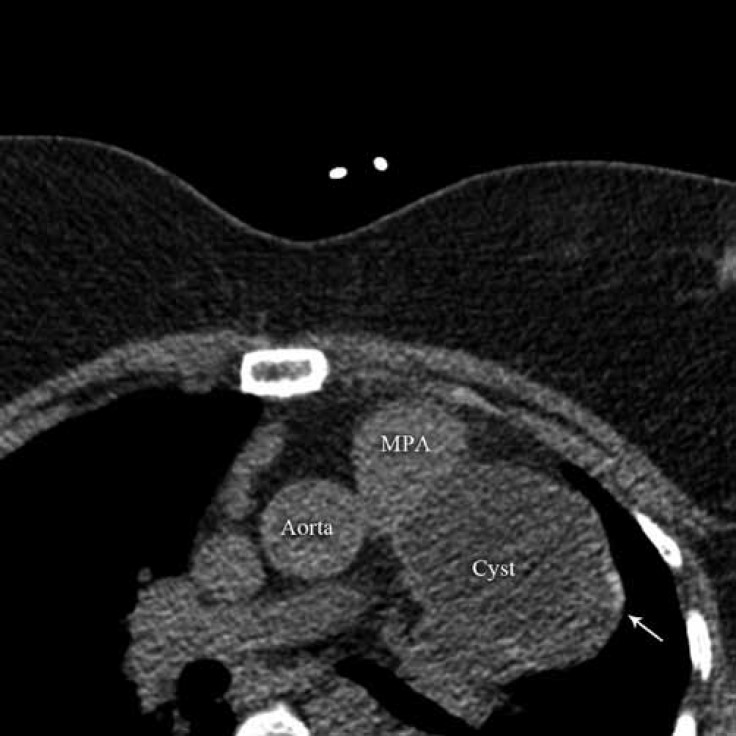
Cross-section of the mediastinum in the computed tomography angiography reveals a large cyst (arrow).

In the emergency department, the patient was hemodynamically stable. In physical examination, her blood pressure was 116/70 mmHg and her pulse rate was 86 beats per minute. Her respiratory rate was within the normal range. She was afebrile. Cardiac examination was normal, but auscultation revealed relatively muffled heart sounds. The examinations of the other organs were grossly normal. Electrocardiogram (ECG) showed no significant ST-T changes. Cardiac troponin T and creatine kinase-myocardial B fraction (CK-MB) were within normal ranges. The patient underwent echocardiography, which revealed normal left ventricular (LV) size and function (ejection fraction = 50% – 55%), no regional wall motion abnormality, mild mitral regurgitation, mild tricuspid regurgitation, pulmonary artery pressure of about 32 mmHg, and normal right ventricular (RV) size and function. A round echolucent well-defined mass (47 × 25 mm) on the base and the mid lateral wall of the LV without septation was seen, which seemed extracardiac. The patient underwent dual-source (2 × 128: 256) multislice computed tomography (CT) angiography of the coronary arteries with spiral method maximum-intensity projection, multiplanar reformation, and volume-rendering reconstruction. CT angiography revealed a large (52 mm) exophytic mass, originating from the lateral wall of the LV with upward growth between the LAD and the left circumflex artery and no LV cavity obliteration (Figure 1 and Figure 2). The LAD was stretched upward by the mass at the proximal portion with significant stenosis. The findings of CT angiography were in favor of fat-containing tumors.

LAD, Left anterior descending artery

MPA, Main pulmonary artery

**Figure 3:**
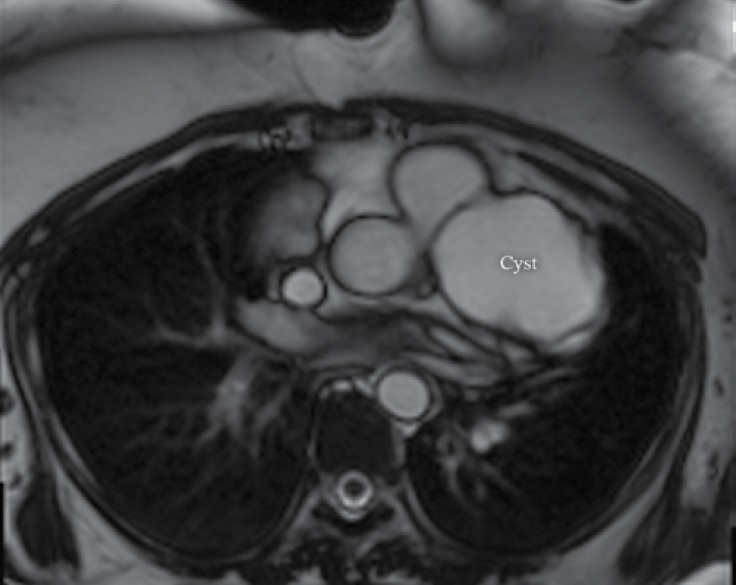
Cardiac magnetic resonance imaging shows a cyst in the patient’s mediastinum.

**Figure 4:**
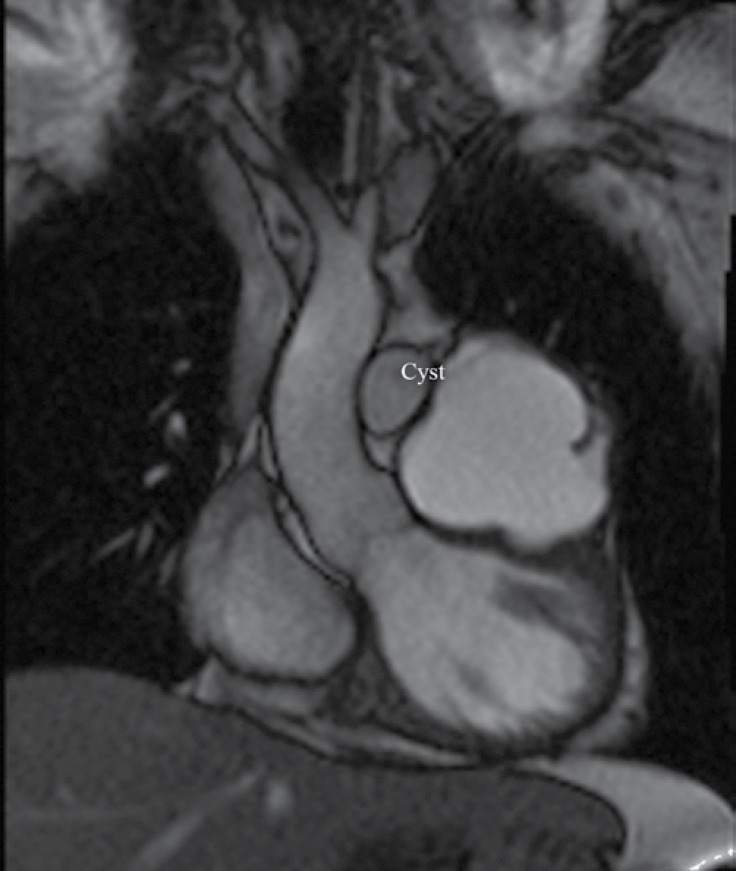
Cardiac magnetic resonance imaging reveals the close proximity of the cyst to vital the parts of the heart.

For preoperative demarcation of the mass and determination of the coronary involvement degree, cardiovascular magnetic resonance imaging and coronary angiography were requested respectively. The former revealed that the mass had cystic components (Figure 3 and Figure 4). 

**Figure 5:**
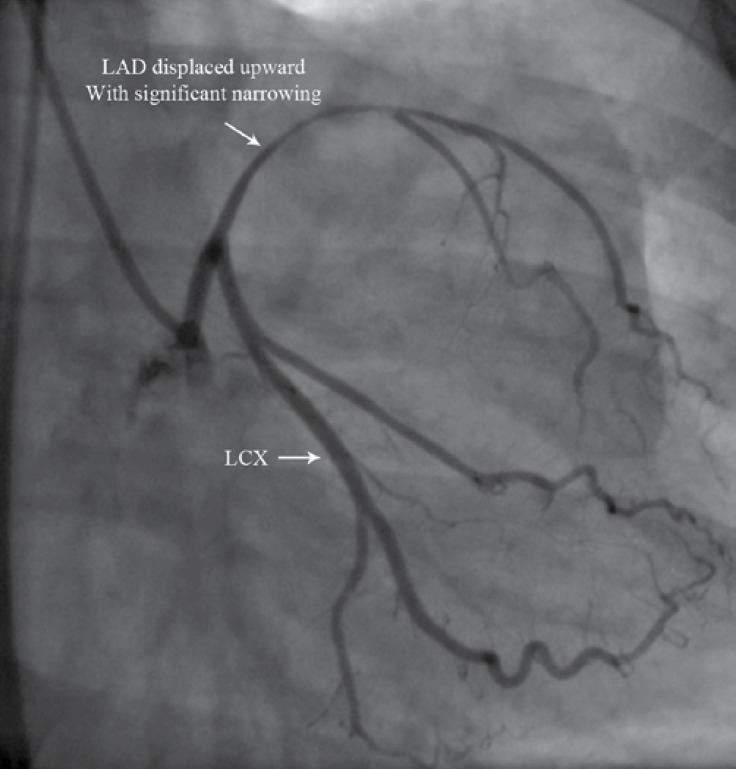
Angiography in the right anterior oblique-caudal projection reveals the upward displacement of the LAD with significant stenosis.

LAD, Left anterior descending artery; LCX, Left circumflex artery

Coronary angiography revealed a normal left main stem and the upward displacement of the LAD with significant compressive narrowing (Figure 5).

**Figure 6:**
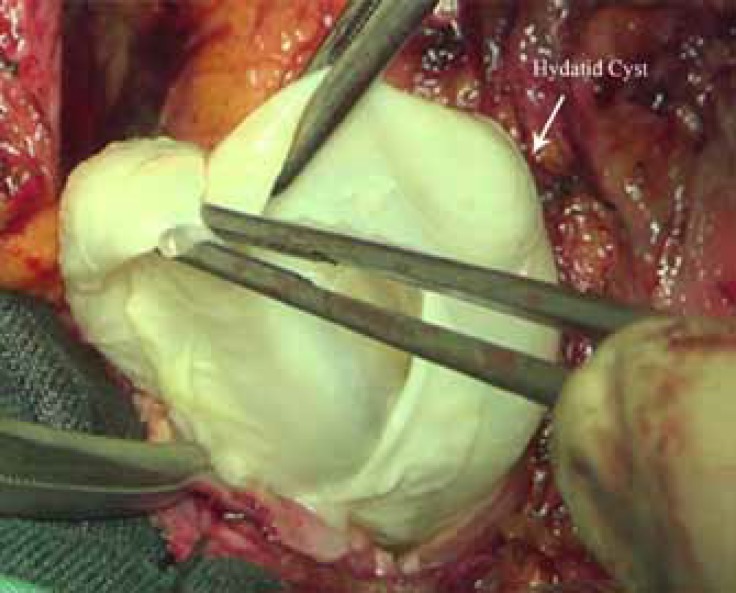
Cyst removal during cardiac surgery.

Our cardiac surgery consultant scheduled the patient for open surgery for mass resection and grafting of the LAD. During surgery, after the incision of the pericardium, we encountered the apparent hydatid cyst entity of the mass with daughter cysts. Hydatid cysts covered the anterolateral surface of the LV with adhesion to the pericardium. The hydatid cysts were removed and marsupialization was done (Figure 6).

Ethanol 96% was used as the scolicidal agent. The left internal mammary artery was harvested and grafted to the beating heart. The patient recovered the surgery uneventfully. Albendazole was administered after consultation with an infectious disease specialist. The imaging of the other organs was negative for hydatid cyst dissemination. Retrospectively, the patient was once more questioned about contact with sources of the disease. She remembered contact with her neighbor’s dogs. The pathology report and immunological assays confirmed the diagnosis. At 6 months’ follow-up (at the time of article preparation), she was asymptomatic and without recurrence.

## Discussion

Although cardiac hydatid cysts are uncommon, they can potentially lead to lethal complications.^9^^, ^^10^ Hydatid disease involves the heart via different routes. Most frequently, the parasite invades the heart through the coronary circulation. Less frequently, cardiac involvement occurs via the pulmonary veins subsequent to pulmonary cyst rupture. The frequent sites of cardiac involvement in hydatid cysts in descending order are the LV (60%), RV (10%), pericardium (7%), pulmonary artery (6%), left atrial appendage (6%), and interventricular septum (4%). The left coronary circulation is more extensive than the right coronary circulation, and the LV mass is greater than the RV. Consequently, the LV is a more suitable location for the parasite.^11^

Patients with cardiac echinococcosis often have no symptoms. When a cyst is adjacent to an important anatomic site, symptoms appear. Vague chest pain is the most common cardiac symptom and angina pectoris is infrequent.^12^ Vague and nonspecific chest pain in cardiac hydatid cysts may lead to the misdiagnosis of coronary artery disease.

Our patient presented with exertional chest pain, which diverted the medical team’s attention to coronary artery disease. The heart is a rare location for hydatid cysts, and isolated cardiac involvement is extremely infrequent. Calcification and septation are usually seen in hydatid cysts, but it is not the rule. In our patient, echocardiography did not show any septation and CT angiography suggested the tumoral nature of the mass with fat content. LAD compression along with the mentioned features listed cardiac cystic tumors in our differential diagnosis. Surgical and pathological findings confirmed hydatid cysts as the etiology. It is important to consider other causes of chest pain in the differential diagnosis, especially in areas with endemic diseases.

Echocardiography and serologic tests are workhorse studies in diagnosing cardiac hydatid cysts. False negative results of serologic tests are possible. In addition, echocardiography is nondiagnostic in some instances. In these cases, CT and magnetic resonance imaging are required to make a crystal-clear diagnosis.^13^^, ^^14^

The World Health Organization’s guidelines for the treatment of cardiac echinococcosis recommend the surgical resection of the cyst as the treatment of choice.^15^ Surgery carries the risk of fluid leakage from the cyst cavity, giving rise to anaphylaxis and the dissemination of infected scolices, which can be minimized by using scolicidal solutions such as iodine, hypertonic saline, methylene blue, and ethanol.^16^^, ^^17^

## Conclusion

To sum up, patients with cardiac hydatid cysts can present with various clinical manifestations even typical angina pectoris. In the differential diagnosis of patients with chest pain, particularly in endemic regions, cardiac hydatid cysts should be considered even for those who do not have a history of hydatid disease. Additionally, it should be noted that negative serology is found in up to 50% of cardiac locations. Increased awareness is essential amongst cardiac physicians and diagnosticians.

## Notes:

***This paper should be cited as: ***Aghajani H, Yazdani S, Forouzan Nia SK, Vahidi H, Aghajani F. Dog Footprint in the Heart. J Teh Univ Heart Ctr 2016;11(4):198-202.
